# Phylogenetic Analysis Reveals a High Prevalence of *Sporothrix brasiliensis* in Feline Sporotrichosis Outbreaks

**DOI:** 10.1371/journal.pntd.0002281

**Published:** 2013-06-20

**Authors:** Anderson Messias Rodrigues, Marcus de Melo Teixeira, G. Sybren de Hoog, Tânia Maria Pacheco Schubach, Sandro Antonio Pereira, Geisa Ferreira Fernandes, Leila Maria Lopes Bezerra, Maria Sueli Felipe, Zoilo Pires de Camargo

**Affiliations:** 1 Departamento de Microbiologia, Imunologia e Parasitologia, Disciplina de Biologia Celular, Universidade Federal de São Paulo (UNIFESP), São Paulo, Brazil; 2 CBS-KNAW Fungal Biodiversity Centre, Utrecht, The Netherlands; 3 Instituto de Ciências Biológicas, Universidade de Brasília (UnB), Brasília, Distrito Federal, Brazil; 4 Instituto de Pesquisa Clínica Evandro Chagas (IPEC), Fundação Oswaldo Cruz, Rio de Janeiro, Brazil; 5 Departamento de Biologia Celular e Genética, Instituto de Biologia Roberto Alcantara Gomes, Universidade do Estado do Rio de Janeiro (UERJ), Rio de Janeiro, Brazil; 6 Ciências Genômicas e Biotecnologia, Universidade Católica de Brasília (UCB), Brasília, Distrito Federal, Brazil; University of California San Diego School of Medicine, United States of America

## Abstract

*Sporothrix schenckii*, previously assumed to be the sole agent of human and animal sporotrichosis, is in fact a species complex. Recently recognized taxa include *S. brasiliensis*, *S. globosa*, *S. mexicana*, and *S. luriei*, in addition to *S. schenckii sensu stricto.* Over the last decades, large epidemics of sporotrichosis occurred in Brazil due to zoonotic transmission, and cats were pointed out as key susceptible hosts. In order to understand the eco-epidemiology of feline sporotrichosis and its role in human sporotrichosis a survey was conducted among symptomatic cats. Prevalence and phylogenetic relationships among feline *Sporothrix* species were investigated by reconstructing their phylogenetic origin using the calmodulin (CAL) and the translation elongation factor-1 alpha (EF1α) loci in strains originated from Rio de Janeiro (RJ, n = 15), Rio Grande do Sul (RS, n = 10), Paraná (PR, n = 4), São Paulo (SP, n = 3) and Minas Gerais (MG, n = 1). Our results showed that *S. brasiliensis* is highly prevalent among cats (96.9%) with sporotrichosis, while *S. schenckii* was identified only once. The genotype of *Sporothrix* from cats was found identical to *S. brasiliensis* from human sources confirming that the disease is transmitted by cats. *Sporothrix brasiliensis* presented low genetic diversity compared to its sister taxon *S. schenckii*. No evidence of recombination in *S. brasiliensis* was found by split decomposition or PHI-test analysis, suggesting that *S. brasiliensis* is a clonal species. Strains recovered in states SP, MG and PR share the genotype of the RJ outbreak, different from the RS clone. The occurrence of separate genotypes among strains indicated that the Brazilian *S. brasiliensis* epidemic has at least two distinct sources. We suggest that cats represent a major host and the main source of cat and human *S. brasiliensis* infections in Brazil.

## Author Summary

Sporotrichosis is a subcutaneous mycosis acquired by traumatic inoculation of soil and plant material contaminated with infectious propagules of the pathogen. The transmission of the disease by cats to other animals and humans occurs by biting or scratching, promoting direct inoculation of yeast cells into host tissue. This may represent an alternative and a successful transmission of the fungus. In order to understand the impact of felines on the epidemiology of sporotrichosis, we evaluated the phenotypic and genotypic features of isolates obtained from animals and humans living in outbreak areas. Although sporotrichosis is caused by a complex of species, in this study we observed that *S. brasiliensis* is the prevalent etiological agent of feline sporotrichosis, having been recovered from 96.9% of the samples. Moreover, this approach allowed us to recognize that isolates from RJ, SP, PR and MG states are genetically similar among them but different from feline isolates recovered from the RS epidemic. Our study brings new insights into the eco-epidemiology of sporotrichosis in Brazil, clarifying the distribution and prevalence of *S. brasiliensis* in feline outbreaks. Knowledge about the source and distribution of the etiological agent between outbreak areas may help to establish public strategies for the containment of the epidemic of sporotrichosis in Brazil.

## Introduction

Mycotic diseases, particularly those caused by dimorphic fungi such as *Sporothrix*, can be considered as an emerging threat to various species of animals. Upon introduction of propagules into the mammalian host, the fungus undergoes a thermodimorphic transition to a yeast-like phase, leading to infections varying between fixed localized cutaneous lesions to severe, disseminated sporotrichosis.

The first connection between *Sporothrix* and animals was made by Lutz and Splendore [1]. Since then sporotrichosis has been reported in dogs, cats, horses, cows, camels, dolphins, goats, mules, birds, pigs, rats, and armadillos, as well as in humans. However, the cat is the animal species most affected by this mycosis [2]. Over the last two decades, Brazil has experienced its largest epidemic of sporotrichosis due to zoonotic transmission, whereby cats were pointed out as key susceptible host. The zoonotic potential of infected cats has been demonstrated by the isolation of *S. schenckii s.l.* from feline skin lesions, nasal, oral cavities, and claw fragments [3], [4].

In contrast to the classical route of infection by *Sporothrix*, where soil and plant material loaded with saprophytic hyphae of the pathogen were the source of contamination [5], transmission of *Sporothrix* spp. by cats to other cats and to humans via direct inoculation of yeast cells represents an alternative and a successful type of dispersal of the disease. The yeast form is more virulent than the mycelial form [6], [7]. Transmission of yeast cells may enhance the appearance of more severe forms of the disease.

Until recently, *S. schenckii* was considered to be the only species causing sporotrichosis. The infection has a worldwide distribution, mainly in tropical and subtropical countries [8]–[10]. The most common clinical manifestations in humans are the lymphocutaneous and fixed forms, but other clinical types, such as a disseminated form, may also occur [11], [12], partly depending on the immune status of the host.

Multilocus sequencing combined with morphological and physiological data support the separation of at least four distinct *Sporothrix* species within the *S. schenckii* complex, uniting the species with high pathogenic potential to mammals. The original taxon *S. schenckii* (Clades IIa and IIb) and the novel species *S. brasiliensis* (Clade I), *S. globosa* (Clade III), and *S. luriei* (Clade VI) todays are referred to as the *S. schenckii* complex [13], while the mildly pathogenic species *S. mexicana* (Clade IV) takes a remote position near the environmental species *S. pallida* [11], [14]–[19]. The *Sporothrix* species differ in their pathogenic potential for mammals [20], [21], their geographical distribution [11], [13], [15], [17], and in their sensitivity to antifungal therapy [22]. All species have been reported from Brazil [11].

Endemic areas of sporotrichosis in Brazil are characterized by poor sanitation, substandard housing and little or no access to health services – a challenge to control and eradication of the disease. The oldest outbreaks of sporotrichosis among humans and cats have been reported in the states of Rio de Janeiro [3], [23], [24] and Rio Grande do Sul [25], [26]. Delayed diagnosis and treatment in cats may lead to a rapid spread of the disease through the community members. The increase in the number of cases in cats is followed by higher numbers of human cases, which constitutes a serious public health problem.

Despite the increasing frequency and severity of cases, the eco-epidemiology of feline sporotrichosis in Brazil is still unknown. The aim of the present study was to determine the distribution and prevalence of *Sporothrix* species among naturally infected felines using phenotypic and molecular phylogenetic approaches.

## Methods

### Isolates and culture conditions

**Figure 1:**
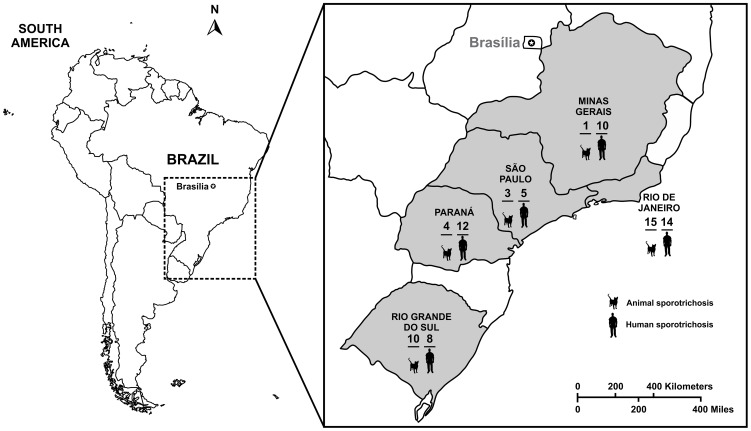
South America map showing sampling localities in Brazil and total number of animals (n = 33) and humans *Sporothrix* spp. **(n = 49) isolates evaluated in Rio de Janeiro, Minas Gerais, São Paulo, Paraná and Rio Grande do Sul.** = 17) outside the gray area and used as control are not shown in the picture.

Thirty three (33) *Sporothrix* isolates from Rio de Janeiro, RJ (n = 15); Rio Grande do Sul, RS (n = 10); Paraná, PR (n = 4); São Paulo, SP (n = 3) and Minas Gerais, MG (n = 1) were obtained from lesions of cats and dogs with sporotrichosis (skin or mucosa lesions) (Fig. 1). Fungal cells were recovered directly from lesions and cultured on Mycosel agar (Difco Laboratories, Detroit, Mich.). Suspected colonies were subcultured on potato dextrose agar (Difco Laboratories, Detroit, Mich.) at room temperature. Isolates were identified phenotypically as *S. schenckii s.l.* As a control, human clinical isolates (n = 66) inside and outside the Brazilian feline outbreaks areas were included in the study (Table 1).

**Table 1:** Strains, species, origin, and GenBank accession numbers of *Sporothrix* spp. isolates used in this study.

					GenBank		
Isolate code	CBS code	Species	Source	Geographic origin	CAL	EF1α	Reference^1^
Ss05	CBS 132985	*Sporothrix brasiliensis*	Feline sporotrichosis	Belo Horizonte, MG, Brazil	KC693830	KC576544	This study
Ss53	CBS 132989	*Sporothrix brasiliensis*	Feline sporotrichosis	Rio Grande, RS, Brazil	KC693846	KC576568	This study
Ss54	CBS 132990	*Sporothrix brasiliensis*	Feline sporotrichosis	Rio Grande, RS, Brazil	JQ041903	KC576569	This study
Ss152	CBS 132995	*Sporothrix brasiliensis*	Feline sporotrichosis	Pelotas, RS, Brazil	KC693865	KC576596	This study
Ss153	CBS 132996	*Sporothrix brasiliensis*	Feline sporotrichosis	Pelotas, RS, Brazil	KC693866	KC576597	This study
Ss154	-	*Sporothrix brasiliensis*	Feline sporotrichosis	Pelotas, RS, Brazil	KC693867	KC576598	This study
Ss155	-	*Sporothrix brasiliensis*	Feline sporotrichosis	Pelotas, RS, Brazil	KC693868	KC576599	This study
Ss156	CBS 132997	*Sporothrix brasiliensis*	Feline sporotrichosis	Pelotas, RS, Brazil	KC693869	KC576600	This study
Ss157	CBS 132998	*Sporothrix brasiliensis*	Feline sporotrichosis	Pelotas, RS, Brazil	KC693870	KC576601	This study
Ss171	CBS 132999	*Sporothrix brasiliensis*	Feline sporotrichosis	Londrina, PR, Brazil	KC693871	KC576602	This study
Ss172	CBS 133000	*Sporothrix brasiliensis*	Feline sporotrichosis	Londrina, PR, Brazil	KC693872	KC576603	This study
Ss173	CBS 133001	*Sporothrix brasiliensis*	Feline sporotrichosis	Londrina, PR, Brazil	KC693873	KC576604	This study
Ss174	CBS 133002	*Sporothrix brasiliensis*	Feline sporotrichosis	Londrina, PR, Brazil	KC693874	KC576605	This study
Ss226	CBS 133003	*Sporothrix brasiliensis*	Feline sporotrichosis	São Paulo, SP, Brazil	KC693875	KC576616	This study
Ss245	CBS 133005	*Sporothrix brasiliensis*	Feline sporotrichosis	Rio de Janeiro, RJ, Brazil	KC693878	KC576619	This study
Ss246	-	*Sporothrix brasiliensis*	Feline sporotrichosis	Rio de Janeiro, RJ, Brazil	KC693879	KC576620	This study
Ss247	CBS 133006	*Sporothrix brasiliensis*	Feline sporotrichosis	Rio de Janeiro, RJ, Brazil	KC693880	KC576621	This study
Ss248	CBS 133007	*Sporothrix brasiliensis*	Feline sporotrichosis	Rio de Janeiro, RJ, Brazil	KC693881	KC576622	This study
Ss249	CBS 133008	*Sporothrix brasiliensis*	Feline sporotrichosis	Rio de Janeiro, RJ, Brazil	KC693882	KC576623	This study
Ss250	CBS 133009	*Sporothrix brasiliensis*	Feline sporotrichosis	Rio de Janeiro, RJ, Brazil	KC693883	KC576624	This study
Ss251	CBS 133010	*Sporothrix brasiliensis*	Feline sporotrichosis	Rio de Janeiro, RJ, Brazil	KC693884	KC576625	This study
Ss252	CBS 133011	*Sporothrix brasiliensis*	Feline sporotrichosis	Rio de Janeiro, RJ, Brazil	KC693885	KC576626	This study
Ss253	CBS 133012	*Sporothrix brasiliensis*	Feline sporotrichosis	Rio de Janeiro, RJ, Brazil	KC693886	KC576627	This study
Ss254	CBS 133013	*Sporothrix brasiliensis*	Feline sporotrichosis	Rio de Janeiro, RJ, Brazil	KC693887	KC576628	This study
Ss255	CBS 133014	*Sporothrix brasiliensis*	Feline sporotrichosis	Rio de Janeiro, RJ, Brazil	KC693888	KC576629	This study
Ss256	CBS 133015	*Sporothrix brasiliensis*	Feline sporotrichosis	Rio de Janeiro, RJ, Brazil	KC693889	KC576630	This study
Ss257	CBS 133016	*Sporothrix brasiliensis*	Feline sporotrichosis	Rio de Janeiro, RJ, Brazil	KC693890	KC576631	This study
Ss258	CBS 133017	*Sporothrix brasiliensis*	Feline sporotrichosis	Rio de Janeiro, RJ, Brazil	KC693891	KC576632	This study
Ss259	CBS 133018	*Sporothrix brasiliensis*	Feline sporotrichosis	Rio de Janeiro, RJ, Brazil	KC693892	KC576633	This study
Ss260	CBS 133019	*Sporothrix brasiliensis*	Feline sporotrichosis	Pelotas, RS, Brazil	KC693893	KC576634	This study
Ss151	CBS 132994	*Sporothrix brasiliensis*	Canine sporotrichosis	Pelotas, RS, Brazil	KC693864	KC576595	This study
Ss227	CBS 133004	*Sporothrix brasiliensis*	Canine sporotrichosis	São Paulo, SP, Brazil	KC693876	KC576617	This study
Ss07	CBS 132986	*Sporothrix brasiliensis*	Human sporotrichosis	Belo Horizonte, MG, Brazil	KC693831	KC576546	This study
Ss08	-	*Sporothrix brasiliensis*	Human sporotrichosis	Belo Horizonte, MG, Brazil	KC693832	KC576547	This study
Ss09	-	*Sporothrix brasiliensis*	Human sporotrichosis	Belo Horizonte, MG, Brazil	KC693833	KC576548	This study
Ss10	CBS 132987	*Sporothrix brasiliensis*	Human sporotrichosis	Belo Horizonte, MG, Brazil	KC693834	KC576549	This study
Ss12	-	*Sporothrix brasiliensis*	Human sporotrichosis	Belo Horizonte, MG, Brazil	KC693835	KC576550	This study
Ss25	CBS 132988	*Sporothrix brasiliensis*	Human sporotrichosis	Curitiba, PR, Brazil	KC693840	KC576556	This study
Ss27	-	*Sporothrix brasiliensis*	Human sporotrichosis	Curitiba, PR, Brazil	JX077111	KC576558	[11]
Ss38	-	*Sporothrix brasiliensis*	Human sporotrichosis	Curitiba, PR, Brazil	KC693844	KC576563	This study
Ss52	-	*Sporothrix brasiliensis*	Human sporotrichosis	São Paulo, SP, Brazil	KC693845	KC576567	This study
Ss55	-	*Sporothrix brasiliensis*	Human sporotrichosis	Rio Grande, RS, Brazil	KC693847	KC576570	This study
Ss56	-	*Sporothrix brasiliensis*	Human sporotrichosis	Rio Grande, RS, Brazil	KC693848	KC576571	This study
Ss62	CBS 132991	*Sporothrix brasiliensis*	Human sporotrichosis	Vila Velha, ES, Brazil	JX077113	KC576572	[11]
Ss69	-	*Sporothrix brasiliensis*	Human sporotrichosis	Rio de Janeiro, RJ, Brazil	KC693849	KC576575	This study
Ss70	-	*Sporothrix brasiliensis*	Human sporotrichosis	Rio de Janeiro, RJ, Brazil	KC693850	KC576576	This study
Ss71	-	*Sporothrix brasiliensis*	Human sporotrichosis	Rio de Janeiro, RJ, Brazil	KC693851	KC576577	This study
Ss72	-	*Sporothrix brasiliensis*	Human sporotrichosis	Rio de Janeiro, RJ, Brazil	KC693852	KC576578	This study
Ss79	-	*Sporothrix brasiliensis*	Human sporotrichosis	Rio de Janeiro, RJ, Brazil	KC693856	KC576582	This study
Ss82	CBS 132992	*Sporothrix brasiliensis*	Human sporotrichosis	Rio de Janeiro, RJ, Brazil	KC693857	KC576584	This study
Ss87	CBS 132993	*Sporothrix brasiliensis*	Human sporotrichosis	Rio de Janeiro, RJ, Brazil	KC693858	KC576585	This study
Ss125	-	*Sporothrix brasiliensis*	Human sporotrichosis	Campinas, SP, Brazil	JX077116	KC576588	[11]
Ss128	-	*Sporothrix brasiliensis*	Human sporotrichosis	Campinas, SP, Brazil	KC693861	KC576589	This study
Ss149	-	*Sporothrix brasiliensis*	Human sporotrichosis	Pelotas, RS, Brazil	KC693862	KC576593	This study
Ss150	-	*Sporothrix brasiliensis*	Human sporotrichosis	Pelotas, RS, Brazil	KC693863	KC576594	This study
CBS 120339^T^	CBS 120339^T^	*Sporothrix brasiliensis*	Human sporotrichosis	Rio de Janeiro, RJ, Brazil	AM116899	KC576606	[19]
IPEC 16919	-	*Sporothrix brasiliensis*	Human sporotrichosis	Rio de Janeiro, RJ, Brazil	AM116898	KC576607	[19]
Ss261	-	*Sporothrix brasiliensis*	Human sporotrichosis	Pelotas, RS, Brazil	KC693894	KC576635	This study
Ss265	CBS 133020	*Sporothrix brasiliensis*	Human sporotrichosis	Uberaba, MG, Brazil	JN204360	KC576636	[12]
Ss01	CBS 132961	*Sporothrix schenckii*	Feline sporotrichosis	São Paulo, SP, Brazil	KC693828	KC576540	This study
Ss02	CBS 132962	*Sporothrix schenckii*	Human sporotrichosis	Porto Alegre, RS, Brazil	KC693829	KC576541	This study
Ss03	CBS 132963	*Sporothrix schenckii*	Human sporotrichosis	Porto Alegre, RS, Brazil	JX077117	KC576542	[11]
Ss04	-	*Sporothrix schenckii*	Human sporotrichosis	Porto Alegre, RS, Brazil	JX077118	KC576543	[11]
Ss13	-	*Sporothrix schenckii*	Human sporotrichosis	Belo Horizonte, MG, Brazil	KC693836	KC576551	This study
Ss15	-	*Sporothrix schenckii*	Human sporotrichosis	Belo Horizonte, MG, Brazil	KC693837	KC576552	This study
Ss17	-	*Sporothrix schenckii*	Human sporotrichosis	Curitiba, PR, Brazil	KC693838	KC576553	This study
Ss20	-	*Sporothrix schenckii*	Human sporotrichosis	Curitiba, PR, Brazil	JX077119	KC576554	[11]
Ss24	-	*Sporothrix schenckii*	Human sporotrichosis	Curitiba, PR, Brazil	KC693839	KC576555	This study
Ss26	CBS 132965	*Sporothrix schenckii*	Human sporotrichosis	Curitiba, PR, Brazil	KC693841	KC576557	This study
Ss28	-	*Sporothrix schenckii*	Human sporotrichosis	Curitiba, PR, Brazil	JX077121	KC576559	[11]
Ss31	-	*Sporothrix schenckii*	Human sporotrichosis	Curitiba, PR, Brazil	JX077122	KC576560	[11]
Ss35	-	*Sporothrix schenckii*	Human sporotrichosis	Curitiba, PR, Brazil	KC693842	KC576561	This study
Ss36	-	*Sporothrix schenckii*	Human sporotrichosis	Curitiba, PR, Brazil	KC693843	KC576562	This study
Ss39	-	*Sporothrix schenckii*	Human sporotrichosis	Curitiba, PR, Brazil	JQ041899	KC576564	This study
Ss63	CBS 132968	*Sporothrix schenckii*	Human sporotrichosis	Vila Velha, ES, Brazil	JX077123	KC576573	[11]
Ss64	-	*Sporothrix schenckii*	Human sporotrichosis	Vila Velha, ES, Brazil	JX077124	KC576574	[11]
Ss73	-	*Sporothrix schenckii*	Human sporotrichosis	Rio de Janeiro, RJ, Brazil	KC693853	KC576579	This study
Ss75	-	*Sporothrix schenckii*	Human sporotrichosis	Rio de Janeiro, RJ, Brazil	KC693854	KC576580	This study
Ss78	-	*Sporothrix schenckii*	Human sporotrichosis	Rio de Janeiro, RJ, Brazil	KC693855	KC576581	This study
Ss80	CBS 132969	*Sporothrix schenckii*	Human sporotrichosis	Rio de Janeiro, RJ, Brazil	JX077125	KC576583	[11]
Ss90	-	*Sporothrix schenckii*	Human sporotrichosis	Rio de Janeiro, RJ, Brazil	KC693859	KC576586	This study
Ss111	CBS 132971	*Sporothrix schenckii*	Human sporotrichosis	São Paulo, SP, Brazil	KC693860	KC576587	This study
Ss143	-	*Sporothrix schenckii*	Human sporotrichosis	Belém, PA, Brazil	JQ041903	KC576592	[11]
CBS 359.36^T^	CBS 359.36^T^	*Sporothrix schenckii*	Human sporotrichosis	USA	AM117437	KC576614	[19]
CBS93872	CBS 93872	*Sporothrix schenckii*	Human sporotrichosis	France	AM490340	KC576637	[17]
Ss06	CBS 132922	*Sporothrix globosa*	Human sporotrichosis	Belo Horizonte, MG, Brazil	JF811336	KC576545	[11]
Ss41	CBS 132923	*Sporothrix globosa*	Human sporotrichosis	Fortaleza, CE, Brazil	JF811337	KC576565	[11]
Ss49	CBS 132924	*Sporothrix globosa*	Human sporotrichosis	Goiânia, GO, Brazil	JF811338	KC576566	[11]
CBS 120340^T^	CBS 120340^T^	*Sporothrix globosa*	Human sporotrichosis	Spain	AM116908	KC576608	[19]
CBS 130104	CBS 130104	*Sporothrix globosa*	Human sporotrichosis	Spain	AM116905	KC576609	[19]
Ss236	CBS 132925	*Sporothrix globosa*	Human sporotrichosis	Minas Gerais, MG, Brazil	KC693877	KC576618	This study
FMR 8598	CBS130116	*Sporothrix globosa*	Human sporotrichosis	Spain	AM116903	KC576638	[19]
CBS 937.72^T^	CBS 937.72^T^	*Sporothrix luriei*	Human sporotrichosis	South Africa	AM747302	KC576615	[18]
Ss132	CBS 132927	*Sporothrix mexicana*	Human sporotrichosis	São Paulo, SP, Brazil	JF811340	KC576590	[11]
Ss133	CBS 132928	*Sporothrix mexicana*	Human sporotrichosis	Recife, PE, Brazil	JF811341	KC576591	[11]
CBS 120342	CBS 120342	*Sporothrix mexicana*	Vegetal	Mexico	AM398392	KC576610	[17]
CBS 120341^T^	CBS 120341^T^	*Sporothrix mexicana*	Soil	Mexico	AM398393	KC576611	[17]
CBS 302.73^T^	CBS 302.73^T^	*Sporothrix pallida*	Soil	United Kingdom	AM398396	KC576612	[17]
CBS 111110	CBS 111110	*Sporothrix pallida*	Insect	Germany	AM398382	KC576613	[17]
CMW 304	CBS 141.36^T^	*Grosmannia serpens*	Environmental	Italy	JN135300	-	[30]
AFTOL-ID 910	CBS 158.74	*Ophiostoma piliferum*	Environmental	Chile	-	DQ471074	[31]

1 Calmodulin literature reference. IPEC, Instituto de Pesquisa Clínica Evandro Chagas, Fiocruz, Brazil; FMR, Facultat de Medicina i Ciències de la Salut, Reus, Spain; CBS, Centraalbureau voor Schimmelcultures, Utrecht, The Netherlands; KMU, Kanazawa Medical University, Ishikawa, Japan; CMW, Culture Collection of the Forestry and Agricultural Biotechnology Institute (FABI); AFTOL, Assembling the Fungal Tree of Life project; NK, not known;

T , type strain. All “Ss” strains belong to the culture collection of Federal University of São Paulo (UNIFESP). MG, Minas Gerais; RS, Rio Grande do Sul; PR, Paraná; SP, São Paulo; RJ, Rio de Janeiro; ES, Espírito Santo; PA, Pará; CE, Ceará; GO, Goiás, PE, Pernambuco.

### Phenotypic characterization

Morphological identification of cultures was performed according to Marimon *et al.* [17], [18] including vegetative growth on PDA media at 30, 35, 37 and 40°C, colony colors on corn meal agar (Difco Laboratories, Detroit, Mich.), assimilation profiles of raffinose, ribitol and sucrose, and microscopic morphology *in vitro*. Growth at different temperatures was measured according to Mesa-Arango *et al.* [27]: the percent growth inhibition (GI) was calculated at 37°C by the following formula [(colony diameter at 30°C – colony diameter at 37°C)/colony diameter at 30°C]×100. The GI was evaluated by analysis of variance/Tukey test using the GraphPad (GraphPad Prism v. 5.00 for Windows, San Diego California USA, www.graphpad.com), considering statistically significant when p<0.05. Observed data were used for taxonomic characterization applying the dichotomous key to species of the *S. schenckii* complex proposed by Marimon *et al.* [18].

### DNA extraction, PCR amplification and DNA sequencing

For molecular analysis, genomic DNA was extracted and purified directly from mycelial colonies following the Fast DNA kit protocol (MP Biomedicals, Vista, CA, USA) with the homogenization step repeated three times with a Precellys 24 instrument (Bertin, Montigny le Bretonneux, France). DNA was quantified with NanoDrop 2000 spectrophotometer (Thermo Fisher Scientific, Wilmington, DE, USA). The calmodulin (CAL) locus region was amplified directly from the genomic DNA by PCR, as described by O'Donnell *et al.* [28], using the degenerate primers CL1 (5′-GAR TWC AAG GAG GCC TTC TC-3′) and CL2A (5′-TTT TTG CAT CAT GAG TTG GAC-3′), which generated an 800-bp amplicon corresponding to exons 3 through 5. The translation elongation factor-1 alpha (EF1α) locus region was amplified using the newly designed primers EF1-F (5′-CTG AGG CTC GTT ACC AGG AG-3′) and EF1-R (5′-CGA CTT GAT GAC ACC GAC AG-3′) which amplified an 850-bp fragment, covering the last exon of this gene, matching the same region evaluated by the consortium Assembling the Fungal Tree of Life (AFTOL).

Thermal cycling conditions were as follows: one cycle of 5 min at 95°C, followed by 35 cycles of 1 min at 95°C, 1 min at 60°C (CAL) or 57°C (EF1α) and 1 min at 72°C, followed by one cycle of 10 min at 72°C.

Amplified products were gel-purified with the Wizard® SV Gel and PCR Clean-Up System (Promega, USA) following the manufacturer instructions. DNA samples were completely sequenced with an ABI 3730 DNA Analyser (Applied Biosystems, Foster City, CA, USA) using BigDye® Terminator v3.1 Cycle Sequencing Kit (Applied Biosystems). The fragments were sequenced on both strands to increase the quality of sequence data and assembled into single sequences via CAP3 using bases with quality of phred ≥30. Sequences were aligned with MAFFT v. 5.667 [29] and retrieved alignments were manually edited in order to avoid mis-paired bases.

### Phylogenetic analysis

Calmodulin sequences deposited at GenBank belonging to the clades of clinical importance in the *S. schenckii* complex (Table 1) were collected and included in the present alignment as reference strains for the phylogenetic distribution. We choose the saprophytic fungus *Grosmannia serpens* (Ophiostomataceae), CBS 141.36 [30] as outgroup for CAL analysis [11]. All *Sporothrix* EF1α sequences used in the phylogenetic analysis were generated in this study (Table 1). The outgroup for the EF1α analysis included the saprophytic fungus *Ophiostoma piliferum*, CBS 158.74 (AFTOL-ID 910) [31]. This species was chosen because the genus *Ophiostoma* (Ophiostomataceae) is considered a close related group to *Sporothrix* species [32].

Phylogenetic analyses were carried out using Neighbor-joining, Maximum Likelihood and Bayesian methods. Neighbor-Joining and Maximum Likelihood trees were constructed using MEGA 5 software [33] and 1000 bootstrap replicates were used to estimate confidence values for individual clades and are shown next to the branches [34]. The evolutionary distances were computed using the Tamura 3-parameters method [35] and the rate variation among sites was modeled with a gamma distribution (shape parameter = 1). For Bayesian analysis by Markov Chain Monte Carlo (MCMC), two independent analyses of four chains each as default were initiated from a random tree and processed for 1.000.000 generations; sample trees were retrieved every 1000 generations. Log-likelihood scores were plotted against its generation number in order to evaluate convergence; samples collected prior to “*burn-in*” (25%) were ignored. The remaining samples were used to determine the distribution of posterior probability values [36]. The posterior probabilities values of generated clades and overall topology of each replicate were compared in order to verify that each consensus tree converged on a similar phylogeny. Phylograms generated by Bayesian analysis were used to represent the phylogenetic distribution and were produced with the help of the Figtree 1.0 software (available at http://tree.bio.ed.ac.uk/software/figtree/).

### Haplotype network

Evolutionary relationships at the intraspecific level were evaluated using haplotype networks in order to visualize differences and diversity among *S. brasiliensis* sequence data. The number and diversity of CAL and EF1α haplotypes were estimated using the software DNAsp v5.0 [37]. Gaps and missing data were excluded in the calculation. Median-joining networks [38] for the CAL and EF1α dataset were obtained and visualized using the software Network 4.610 (available at www.fluxus-engineering.com).

### Recombination event detection

Evidence of recombination in *S. brasiliensis* population isolated from animals and humans samples was inferred by the split decomposition method [39], implemented by the Splitstrees4 software, version 4.8 [40] which is used to identify branching ambiguities attributable to recombination events. The presence of recombination networks can be detected by bridges between members of the genetically isolated groups. Each isolated group will have an independent branch, showing that it does not share genetic material with the others. This analysis allowed the assessment of recombination possibilities within and between the seven phylogenetic groups considered.

The PHI-test incorporated in the SplitsTree software [40] was used to test signals of recombination (p<0.05, significant evidence of recombination). The test is proven to be a robust calculation and no previous knowledge about population history, recombination rate, mutation rate and rate heterogeneity across sites [41] is necessary. Although large splits in networks do not necessarily imply recombination, split decomposition networks in conjunction with the PHI-test can easily detect which sequences in a given data set contribute the most to the recombination signal [42]. The PHI-test is repeated after possible recombinants are deleted from the alignment until p>0.05 (no evidence of recombination). Also, DNAsp v5.1 [43] was used to evaluate minimum number of recombination events in the history and haplotypic diversity of *S. brasiliensis* population. The software computes the recombination parameter R = 4Nr, where N is the population size and r is the recombination rate per sequence -or between adjacent sites [44].

### Ethics statement

The animals included in this study were examined by a veterinarian with experience in small animal internal medicine. The procedures performed in these animals were approved by the Ethics in Research Committee (CEUA) of the FIOCRUZ, Rio de Janeiro, Brazil, under license number L-041/06.

## Results

**Figure 2:**
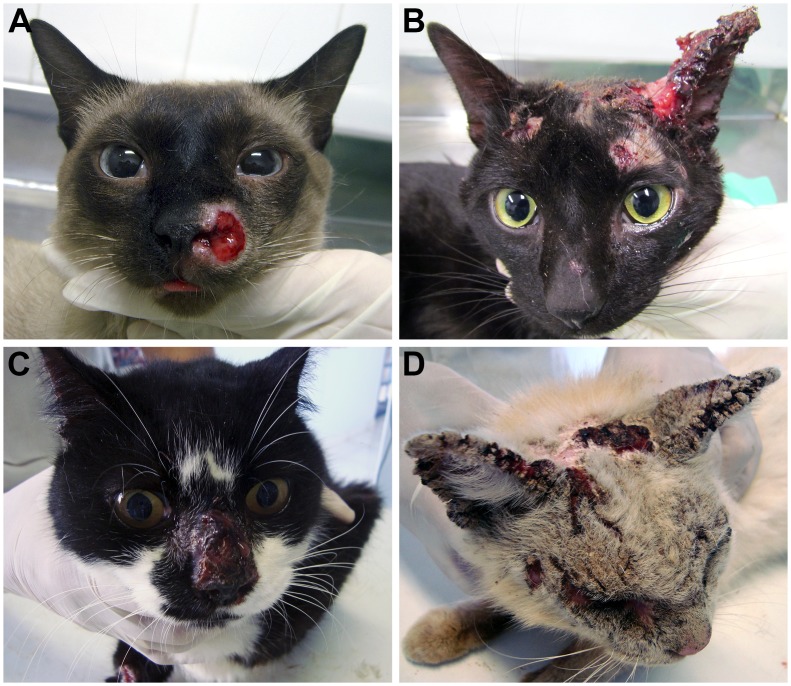
Clinical aspects of feline sporotrichosis in Brazil. **Cats presenting ulcerated cutaneous lesions in the cephalic region.** (A) and (B) felines from Rio de Janeiro; (C) and (D) felines from Paraná.

Our study included indoor and feral cats from five different geographic regions in Brazil (RJ, RS, MG, SP, and PR). Diagnosis of sporotrichosis was performed by the clinical evaluation of skin lesions and confirmed by isolation of the pathogen. The suspected colonies of *Sporothrix* species were grown on Mycosel agar until purification of the pathogen. The fungus was easily isolated from material from the nasal, oral mucosa and skin lesions. Lesions in the cephalic region and/or respiratory tract were observed in most of the animals (Fig. 2).

Phenotypic characterization, i.e. growth at various temperatures, macroscopic and microscopic features, and carbohydrate assimilation, yielded data similar to those found for the reference strains of *S. brasiliensis* (CBS 120339) and *S. schenckii* (CBS 359.36) reported by Marimon *et al.* [17]. Among the 33 strains of *Sporothrix* isolated from cats (n = 31) and dogs (n = 2) from different geographic regions of Brazil, 32 belonged to *S. brasiliensis* (96.9%) and 1 to *S. schenckii* (3%). These phenotypic results showed that *S. brasiliensis* is highly prevalent among cats with sporotrichosis. The two isolates recovered of canine sporotrichosis (CBS 132994 and CBS 133004 from RS and SP, respectively) were identified as *S. brasiliensis*.

Using CL1 and CL2A primers we amplified 800 bp of the CAL locus. The complete alignment included 100 strains. Aligned sequences of CAL were 727 bp long, including 366 invariable characters, 214 variable parsimony-informative (29.43%), and 125 singletons. Comparison with sequences available at GenBank revealed a match of 99–100% with the type strain of *S. brasiliensis* (CBS 120339, AM116899) corroborating our phenotypic data. The single isolate of *S. schenckii* (CBS 132961) matched 99% with the *S. schenckii s. str.* strain (FMR 8678, AM117446) from Argentina.

**Figure 3:**
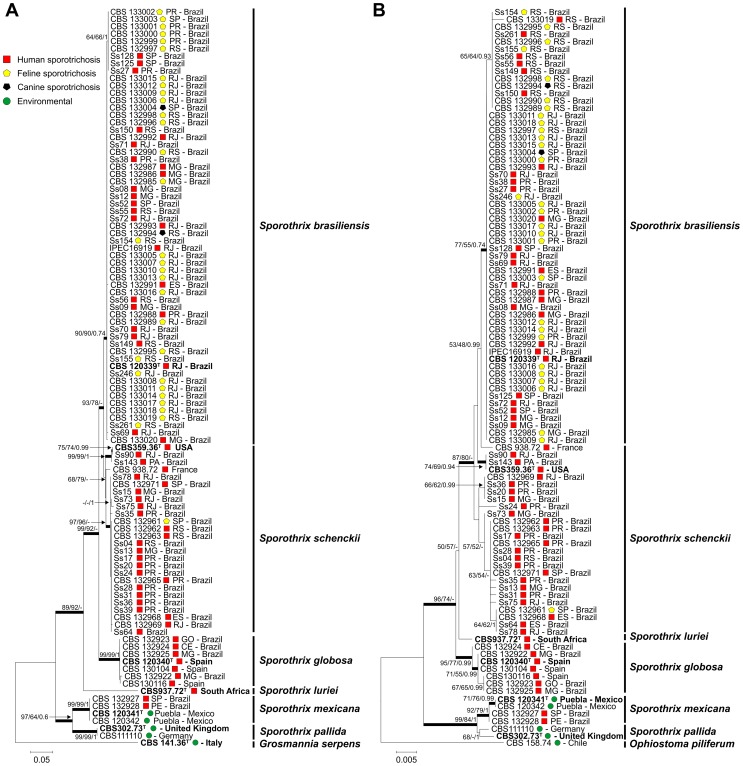
Phylogenetic trees generated by Neighbor-joining, Maximum Likelihood and Bayesian analysis using partial nucleotide sequences of the calmodulin-encoding gene (A) and the translation elongation factor-1 alpha (EF1α) locus region (B). Bootstrap and posterior probabilities values were added to respective branches (NJ/ML/BI). Each species are indicated at each respective position at the phylogenetic tree. Calmodulin and EF1α accessions number are indicated in the Table 1.

Phylogenetic analysis of isolates from cats and dogs revealed that *S. brasiliensis* is the prevalent species (32/33); only a single isolate clustered with *S. schenckii s. str*. The clade of pathogenic *Sporothrix* species was well supported with high bootstrap and posterior probability values. The *S. brasiliensis* isolates recovered from animal sources clustered in a single branch together with clinical isolates, indicating that they belonged to the same genotypes and confirming that the disease is transmitted by cats. A cryptic branch was observed in the *S. brasiliensis* clade composed of the isolates Ss27, Ss125, Ss128, CBS 132997, CBS 132999, CBS 133000, CBS 133001 CBS 133002 and CBS 133003, supported by bootstrap and posterior probabilities values (64/66/1) (Fig. 3A).

*Sporothrix brasiliensis* presented low genetic diversity compared to its sister taxon *S. schenckii* when CAL was used as a marker. Elongation factor (EF1α) was used as marker to assess the genetic diversity in the species. All isolates presented similar fragments of 850 bp of the EF1α locus which were amplified and sequenced with primers EF1-F and EF1-R. Aligned sequences of EF1α were 707 bp long, including 639 invariable characters, 34 variable parsimony-informative (5.08%), and 33 singletons. The 100 OTUs were distributed into 7 main groups (Fig. 3B), which were congruent with the CAL phylogeny.

Judging from the EF1α dataset, the *S. brasiliensis* isolates recovered from animal sources in RJ and RS clustered in two branches with human clinical isolates from the same states, indicating two epidemics with distinct genotypes are concerned (Fig. 3B). *Sporothrix brasiliensis* presented low genetic diversity in EF1α, in accordance with results obtained for the CAL locus.

**Figure 4:**
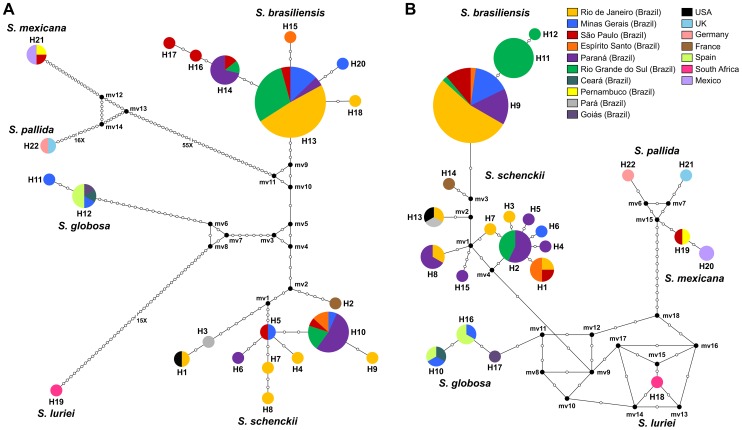
Median-joining haplotype network of *Sporothrix schenckii* complex isolates based on partial nucleotide sequences of the calmodulin-encoding gene (A) and the translation elongation factor-1 alpha (EF1α) loci regions (B). The EF1α haplotype showed a clear intraspecific separation resultant from a nucleotide transition from A to G, between *S. brasiliensis* isolates recovered from Rio de Janeiro (H9) and Rio Grande do Sul (H11 and H12) feline epidemics. The size of the circumference is proportional to the haplotype frequency. Black dots (median vectors) are hypothetical missing intermediates. Calmodulin and EF1α haplotypes are detailed in the Table S2.

The haplotype diversity of *S. brasiliensis* species was assessed using the DNASp software. Only 7 haplotypes for CAL (Fig. 4A) and 3 haplotypes for EF1α (Fig. 4B) were found. The low values of haplotype (Hd_CAL_ = 0.36 and Hd_EF1α_ = 0.37) and nucleotide diversities (π_CAL_ = 0.00152 and π_EF1α_ = 0.00062) lead us to hypothesize that this species is clonal (Table S1). Geographical separation between the RJ and RS epidemics for the EF1α locus was clear. The median-joining network based on the EF1α haplotype showed an intraspecific separation (Fig. 4B, haplotypes H11 and H12) resulting from a nucleotide transition from A to G, between isolates from RJ and RS epidemics (Table S2). The average divergence between *S. brasiliensis* and its sister species *S. schenckii* is much higher, suggesting that the species experienced different evolutionary processes.

**Figure 5:**
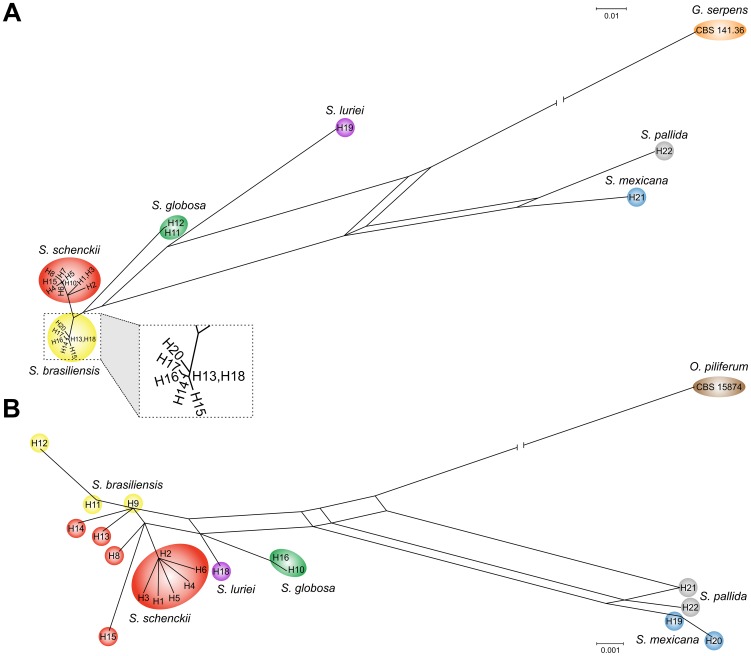
Split decomposition analysis of the *Sporothrix brasiliensis* isolates from zoonotic epidemic outbreaks in different geographic regions in Brazil according to sequences of the calmodulin-encoding gene (A) and the translation elongation factor-1 alpha (EF1α) locus region (B). The inset Box represents the *S. brasiliensis* species alone, showing the absence of recombination possibilities within this species. The absence of reticulated phylogenetic structure in the *S. brasiliensis* haplotypes suggests a clonality spread of this species among human, cats and dogs in Brazil for both loci.

Recombination analysis of *S. brasiliensis* was first assessed by split decomposition method and no networks linking different isolates were observed in both datasets (Fig. 5), in agreement with the concept of clonal species. Also PHI-test analysis showed no evidence of recombination (p_CAL_ = 0.757 and p_EF1α_ = 0.903), and no recombination events were detected by DNAsp5 software. Taken together, these analyses indicated that *S. brasiliensis* is a clonal species.

**Figure 6:**
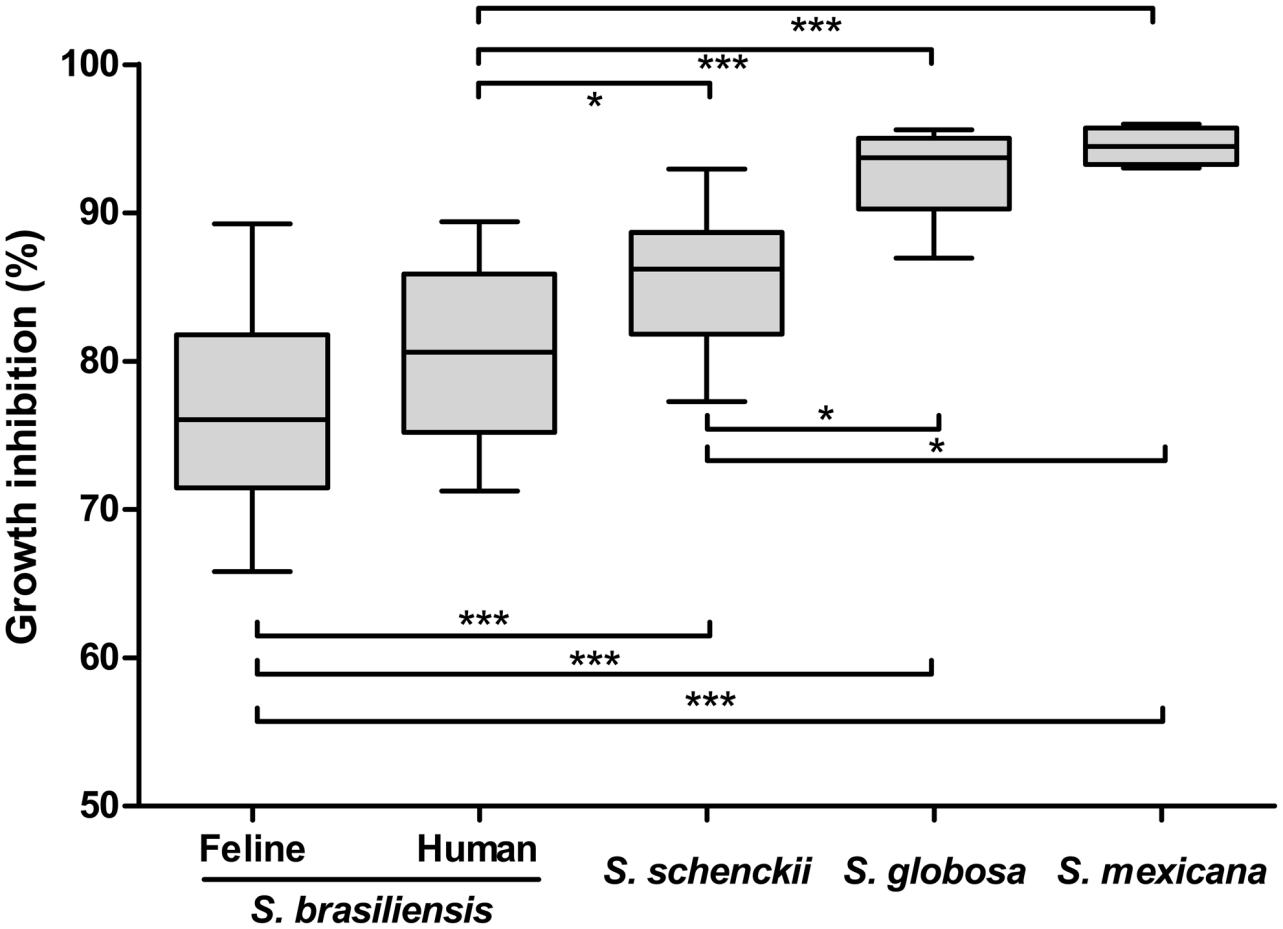
*In vitro* temperature fitness in the *Sporothrix* species. **Growth inhibition at 37°C compared to 30°C incubation.** *S. brasiliensis* from feline (n = 30) or human source (n = 27) are more resistant to heat incubation and differ statistically when compared to *S. schenckii* (n = 25), *S. globosa* (n = 7) and *S. mexicana* (n = 4). Statistical significance in one-way ANOVAs followed by Tukey's tests: * p<0.05, *** p<0.0001. The line in the boxes and upper and lower bars show the median, maximum and minimum values, respectively. Isolates were not compared at superior temperature (38–40°C) due to low growth observed to *S. globosa* and *S. mexicana*. No isolate were able to growth at 40°C.

Aiming to evaluate possible phenotypic characteristics that explain the success of this pathogen adaptation to the feline host we evaluated the thermal resistance of strains of clinical interest (human and animal) and environmental strains. Strains of *S. brasiliensis* from feline origin (n = 30) showed highest temperature tolerance, being inhibited 77.1±6.32% on average at 37°C (Fig. 6). The group differed statistically from other species evaluated herein (*S. schenckii s. str.*, *S. globosa*, and *S. mexicana*), suggesting that this factor may confer advantage during the process of infection in the feline host.

### Supporting information

Supplementary information reported in this section is complementary to the results and describe the genetic diversity of the *Sporothrix* isolates.

## Discussion

Epidemiology of fungal infections can be influenced by several factors, including: (i) biological factors such as fungal virulence and host resistance, (ii) ecological factors such as temperature, atmospheric humidity, ultraviolet radiation, geological conditions, and inter-relationships with other living beings, and (iii) socio-economic factors such as poverty, sanitation, clothing, profession, prophylactic habits and population migrations. In the Brazilian epidemic of feline sporotrichosis a combination of a highly virulent fungus and a susceptible host coupled to low sanitary conditions in the suburbs has made the state of RJ a highly endemic area of this mycosis among animals and humans. The epidemic proportions are noted only since the last two decades.

Little is known about the eco-epidemiology of feline sporotrichosis and its impact on the epidemiology of human sporotrichosis. Cats play a significant role in outbreak areas of sporotrichosis such as RJ and RS. Classically, humans can acquire sporotrichosis by cat scratches or bites, the reason why cats are considered important source of infection in the spread of the disease. In our study we found that *S. brasiliensis* is the prevalent etiological agent of feline sporotrichosis in Brazil. Among cats, *S. brasiliensis* was identified in a total of 96.9% of the samples, by isolation of the pathogen from lesions and posterior phenotypic and molecular characterization.

Interestingly, a correlation between cat outbreaks and prevalence of *S. brasiliensis* among humans was found in the same geographic area, such as in RJ (Table 1). This fact matches with our hypothesis that outbreaks among cats directly influence the prevalence of *S. brasiliensis* in human cases of sporotrichosis in the same geographic area. A similar situation was observed in the state of RS where *S. brasiliensis* was isolated with high frequency from cats as well as from humans.

Marimon *et al.* [17] analyzed 127 *Sporothrix* isolates using the calmodulin locus and five major clades (I–V) were recognized. The maximum likelihood, neighbor-joining and Bayesian analyses based on the calmodulin (Fig. 3A) or EF1α (Fig. 3B) loci placed our animal *Sporothrix* isolates in Clade I (*S. brasiliensis*) composed of clinical samples from the RJ State epidemic, with strong bootstrap and posterior probability support. All pathogenic *Sporothrix* species are known to occur in Brazil [11], but *S. brasiliensis* is relatively frequent among feline sporotrichosis outbreaks.

The geographic origin of *S. brasiliensis* of the Brazilian epidemic is difficult to establish. At least two distinct genotypes occur: one in RS and another in RJ. The latter is the oldest and longest recorded in the literature [3], [4], [23], [24]. Our data show that humans and animals infected in the RS epidemic harbor the same *S. brasiliensis* genotype, which is distinct from the one of the RJ epidemic. The RJ genotype is also present in the recent outbreaks in PR, MG and SP, which suggests spread of *S. brasiliensis* from RJ. Additionally, our results showed absence of recombination events in the CAL and EF1α loci, demonstrating that *S. brasiliensis* is a clonal species. Despite a recent indication of intraspecific variability within the species *S. brasiliensis* using RAPD [45] we believe that this phenomenon is not frequent or strong enough to break the prevalent pattern of clonal population structure, i.e., recombination or scarce exchange of genetic material may occur in some point of the evolutionary course of the pathogen life without compromise or affect its population structure. This hypothesis has been discussed by Tibayrenc and Ayala [46] through different group of pathogens including fungi.

The existence of clonal populations has repeatedly been proven in fungal pathogens [47]–[50], although most of these species are surmised to have occasional sexuality in any phase of their life cycle. Under permissive conditions, most fungi reproduce very effectively by asexual propagation. Sexual reproduction provides advantages to the pathogen under adverse conditions, generating suitable genotypes that enhance survival. Many fungal epidemics are driven by populations showing low levels of genetic diversity, as demonstrated in *Penicillium marneffei* [51], [52], *Cryptococcus gattii* [53], [54] and *Batrachochytrium dendrobatidis* [55]. Also feline and human sporotrichosis in Brazil caused by *S. brasiliensis* is driven by the spread of a clonal species. In contrast, outbreaks of other human pathogens such as *Coccidioides immitis* [56]–[58] and *Paracoccidioides brasiliensis* [59]–[61], spread by a diversity of genotypes.

The ecological aspects of the pathogenic species within the genus *Sporothrix* needs to be reevaluated, and this information can be crucial to find the source of *S. brasiliensis* in nature. Classically, soil [5], thorny plants [62], *Sphagnum* moss [63]–[65] and hay [66] have been pointed as source of *S. schenckii s.l.* To date, just a single environmental isolate (FMR 8337) of *S. brasiliensis* was isolated and reported from domiciliary dust in Brazil [17], [19]. Distant relatives of *Sporothrix* in the fungal order Ophiostomatales are mainly associates of bark beetles on woody plants [67], [68]. Zhou *et al.* [13] demonstrated that different ecologies are corroborated by phylogenetic separation.

It is challenging to obtain environmental isolates of *S. brasiliensis*, and the low number of subjects contaminated with propagules from soil or woody plants is indeed low compared to the high occurrence in warm-blooded hosts [3], [69], [70]. This suggests successful transmission among animals (cat-cat and cat-humans). This scenario is different from epidemics occurring in South Africa [71], [72], India [10], [73], the USA [63], [64], Australia [66], [74], China [75], and Japan [76], where patients are mainly infected through soil and decaying wood. Possibly the Brazilian epidemics of *S. brasiliensis* are related to the emergence of a pathogenic clone front of a highly susceptible feline host, rather than to an increase in population size of *S. brasiliensis* in nature. This is corroborated by the high degree of virulence observed in naturally infected cats in the outbreak area [24], as well as demonstrated in a murine model [21]. Besides that, we do not discharge the hypothesis that the emergence of pathogenicity could also be attributed to a recent host-shift from an unknown host to cats as discussed in other groups of pathogens [77]–[79]. Feral cats present a great potential to spread the disease in a short period of time due to their mobility and digging behavior, whereas dispersal from soil or vegetable remains is ineffective.

Classically, accumulation of mutations in fungal populations can lead to speciation processes. However, rapid emergence of a new, highly virulent pathogen which is able to explore different ecological niches may result from other processes than those observed in natural selection. In many plant-pathogenic fungi, such as *Fusarium* and *Alternaria*, pathogenicity is determined by mobile, dispensable small chromosomes [80], [81]. Genetic processes such as hybridization of two distinct, sympatric species [82], parasexual recombination [83], [84] or mechanisms of inactivation/activation of virulence genes by insertion of transposons [85] can also drive the emergence of pathogenicity. Hybridization is one of the possible mechanisms of emergence of phytopathogenic fungi [86], [87] as well as fungi pathogenic to animals [88]. It has also been discussed in the genus *Ophiostoma*, which is phylogenetically related genus to *Sporothrix* [89]. All these genetic processes, alone or in combination, may be the reason of the emergence of virulence in the species *S. brasiliensis*. The lack of variation in the populations of *S. brasiliensis* also may be the result of a strong selective pressure imposed by the feline host. Presence of opposite mating types and sexual reproduction leads to genetic recombination and may increase fitness and widen host ranges. So far, no evidence of sexual recombination was demonstrated experimentally for the species from the *S. schenckii* complex and this fact, combined with the hostile selective pressure of the cats may provide possible explanations for the lack of diversity in *S. brasiliensis.*

The association of *S. brasiliensis* with cats may play an important role in the evolution and spread of this pathogen. The interaction between cats and *S. brasiliensis* is not an exclusive relationship, since *S. schenckii s. str.* was also found in the feline host. However, *S. brasiliensis* has become predominant in this host within less than a decade, indirectly indicating a recent adaptation to the conditions of the feline body. Therefore, cats represent a natural habitat for *S. brasiliensis*. In contrast to the situation in opportunistic fungi, *Sporothrix* species are able to escape from the host and be transmitted to the next host, which is one of the hallmarks of a pathogen. Transmission is either direct during fights, or indirect, the fungus returning to soil after the cat has died.

Given the role of the mammal host in *Sporothrix* evolution, variance in fitness between clonal lineages of *S. brasiliensis* is expected to lead to populations that are better adapted to host conditions. For example, the body temperature of the feral cat *Felis catus* is around 38–39°C, depending on its activity [90]. Interestingly, *S. brasiliensis* has the best rate of vegetative growth when incubated at 37°C, followed by *S. schenckii s. str.* (Fig. 6). Remaining species of *Sporothrix* such as *S. globosa* and *S. mexicana* appear to be more sensitive to temperature, having a maximum around 35°C. The cat's body temperature could be considered an important selective pressure event, selecting thermo-resistant strains during sporotrichosis outbreak episodes. Transmission of *S. brasiliensis* by cats promotes inoculation into human hosts of yeast cells of rather than of hyphae and conidia, yeast cells having been reported to be more virulent [6].

The endotherm developed by mammals is a natural defense mechanism against pathogens [91]–[93], and in our study this factor appears to restrict the occurrence of species of the *S. schenckii* complex that are sensitive to temperatures above 35–37°C [11], [17].

Another important factor in understanding the success of the epidemic of sporotrichosis among cats in RJ, has a socio-economic character. Most cat owners are living in neglected areas and many abandon dead animals in the street [94], favoring contact with other feral cats, or simply bury their pets after death in their backyard or in nearby wastelands. This directly allows the return of the agent into the environment, increasing outbreak risks of the pathogen, and enhancing the spread of the clonal species. In an epidemic scenario, domestic pets such as cats and dogs are the first animals to become infected with the fungus. Subsequently human cases of sporotrichosis are likely to emerge. Thus, we believe that cats can act as sentinel animals for epidemiological services, and its notification should be compulsory by regulatory agencies as the Centers for Zoonosis Control. The predominance of a species that is highly virulent to humans and animals requires fast implementation of public health policies to contain the epidemic, lowering harmful effects to the population.

## Supporting Information

Table S1 Nucleotide diversity (%π) and haplotype diversity (1 – Σfi^2^) from Brazilian clinical isolates belonging to the *Sporothrix schenckii* complex.
(DOC)

Table S2 Identification of the haplotypes in the *Sporothrix* species according to the calmodulin (CAL) or elongation factor (EF1-α) loci.
(DOC)
