# Supplementary material for: Phylogenetic Analysis Reveals a High Prevalence of Sporothrix brasiliensis in Feline Sporotrichosis Outbreaks
Source: PLoS Negl Trop Dis. 2013 Jun 20;7(6):e2281. doi: 10.1371/journal.pntd.0002281 (PMC3688539; doi:10.1371/journal.pntd.0002281)
Supplement: Table S1 — Nucleotide diversity (%π) and haplotype diversity (1 – Σfi2) from Brazilian clinical isolates belonging to the Sporothrix schenckii complex. (DOC) [file pntd.0002281.s001.doc]

**Table S1:** Nucleotide diversity (%π) and haplotype diversity (1 − Σfi^2^) from Brazilian clinical isolates belonging to the *Sporothrix schenckii* complex.

|  |  | **Number of haplotypes (H)** | | **Haplotype diversity (Hd)** | | **Nucleotide diversity (π)** | |
| --- | --- | --- | --- | --- | --- | --- | --- |
| **Species** | **Sample size (n)** | **CAL** | **EF** | **CAL** | **EF** | **CAL** | **EF** |
| *Sporothrix brasiliensis* | 59 | 8* | 3 | 0,36 | 0,37 | 0,00152 | 0,00062 |
| *Sporothrix schenckii* | 26 | 13 | 11 | 0,84 | 0,86 | 0,00842 | 0,00390 |
| Overall | 85 | 20 | 14 | 0,67 | 0,68 | 0,01688 | 0,00377 |

*The *Sporothrix brasiliensis* dataset present one extra haplotype when evaluated alone, without the others species in the alignment. This difference is due to the exclusion of gaps and missing data in the analysis.
