# Supplementary material for: Phylogenetic Analysis Reveals a High Prevalence of Sporothrix brasiliensis in Feline Sporotrichosis Outbreaks
Source: PLoS Negl Trop Dis. 2013 Jun 20;7(6):e2281. doi: 10.1371/journal.pntd.0002281 (PMC3688539; doi:10.1371/journal.pntd.0002281)
Supplement: Table S2 — Identification of the haplotypes in the Sporothrix species according to the calmodulin (CAL) or elongation factor (EF1-α) loci. (DOC) [file pntd.0002281.s002.doc]

**Table S2:** Identification of the haplotypes in the *Sporothrix* species according to the calmodulin (CAL) or elongation factor (EF1-α) loci.

| **Isolate** | **CBS code** | **Species** | **Geographic origin** | **Geographic origin - Region** | **CAL Hap** | **EF1-α Hap** |
| --- | --- | --- | --- | --- | --- | --- |
| Ss01 | CBS 132961 | *S. schenckii* | São Paulo, SP, Brazil | Southeast | Hap10 | Hap01 |
| Ss02 | CBS 132962 | *S. schenckii* | Porto Alegre, RS, Brazil | South | Hap10 | Hap02 |
| Ss03 | CBS 132963 | *S. schenckii* | Porto Alegre, RS, Brazil | South | Hap10 | Hap02 |
| Ss04 | - | *S. schenckii* | Porto Alegre, RS, Brazil | South | Hap10 | Hap02 |
| Ss05 | CBS 132985 | *S. brasiliensis* | Belo Horizonte, MG, Brazil | Southeast | Hap13 | Hap09 |
| Ss06 | CBS 132922 | *S. globosa* | Belo Horizonte, MG, Brazil | Southeast | Hap11 | Hap16 |
| Ss07 | CBS 132986 | *S. brasiliensis* | Belo Horizonte, MG, Brazil | Southeast | Hap13 | Hap09 |
| Ss08 | - | *S. brasiliensis* | Belo Horizonte, MG, Brazil | Southeast | Hap13 | Hap09 |
| Ss09 | - | *S. brasiliensis* | Belo Horizonte, MG, Brazil | Southeast | Hap13 | Hap09 |
| Ss10 | CBS 132987 | *S. brasiliensis* | Belo Horizonte, MG, Brazil | Southeast | Hap13 | Hap09 |
| Ss12 | - | *S. brasiliensis* | Belo Horizonte, MG, Brazil | Southeast | Hap13 | Hap09 |
| Ss13 | - | *S. schenckii* | Belo Horizonte, MG, Brazil | Southeast | Hap10 | Hap06 |
| Ss15 | - | *S. schenckii* | Belo Horizonte, MG, Brazil | Southeast | Hap05 | Hap08 |
| Ss17 | - | *S. schenckii* | Curitiba, PR, Brazil | South | Hap10 | Hap02 |
| Ss20 | - | *S. schenckii* | Curitiba, PR, Brazil | South | Hap10 | Hap08 |
| Ss24 | - | *S. schenckii* | Curitiba, PR, Brazil | South | Hap10 | Hap15 |
| Ss25 | CBS 132988 | *S. brasiliensis* | Curitiba, PR, Brazil | South | Hap13 | Hap09 |
| Ss26 | CBS 132965 | *S. schenckii* | Curitiba, PR, Brazil | South | Hap10 | Hap02 |
| Ss27 | - | *S. brasiliensis* | Curitiba, PR, Brazil | South | Hap14 | Hap09 |
| Ss28 | - | *S. schenckii* | Curitiba, PR, Brazil | South | Hap10 | Hap02 |
| Ss31 | - | *S. schenckii* | Curitiba, PR, Brazil | South | Hap10 | Hap04 |
| Ss35 | - | *S. schenckii* | Curitiba, PR, Brazil | South | Hap06 | Hap05 |
| Ss36 | - | *S. schenckii* | Curitiba, PR, Brazil | South | Hap10 | Hap08 |
| Ss38 | - | *S. brasiliensis* | Curitiba, PR, Brazil | South | Hap13 | Hap09 |
| Ss39 | - | *S. schenckii* | Curitiba, PR, Brazil | South | Hap10 | Hap02 |
| Ss41 | CBS 132923 | *S. globosa* | Fortaleza, CE, Brazil | Northeast | Hap12 | Hap10 |
| Ss49 | CBS 132924 | *S. globosa* | Goiânia, GO, Brazil | Midwest | Hap12 | Hap17 |
| Ss52 | - | *S. brasiliensis* | São Paulo, SP, Brazil | Southeast | Hap13 | Hap09 |
| Ss53 | CBS 132989 | *S. brasiliensis* | Rio Grande, RS, Brazil | South | Hap13 | Hap11 |
| Ss54 | CBS 132990 | *S. brasiliensis* | Rio Grande, RS, Brazil | South | Hap13 | Hap11 |
| Ss55 | - | *S. brasiliensis* | Rio Grande, RS, Brazil | South | Hap13 | Hap11 |
| Ss56 | - | *S. brasiliensis* | Rio Grande, RS, Brazil | South | Hap13 | Hap11 |
| Ss62 | CBS 132991 | *S. brasiliensis* | Vila Velha, ES, Brazil | Southeast | Hap15 | Hap09 |
| Ss63 | CBS 132968 | *S. schenckii* | Vila Velha, ES, Brazil | Southeast | Hap10 | Hap01 |
| Ss64 | - | *S. schenckii* | Vila Velha, ES, Brazil | Southeast | Hap10 | Hap01 |
| Ss69 | - | *S. brasiliensis* | Rio de Janeiro, RJ, Brazil | Southeast | Hap18 | Hap09 |
| Ss70 | - | *S. brasiliensis* | Rio de Janeiro, RJ, Brazil | Southeast | Hap13 | Hap09 |
| Ss71 | - | *S. brasiliensis* | Rio de Janeiro, RJ, Brazil | Southeast | Hap13 | Hap09 |
| Ss72 | - | *S. brasiliensis* | Rio de Janeiro, RJ, Brazil | Southeast | Hap13 | Hap09 |
| Ss73 | - | *S. schenckii* | Rio de Janeiro, RJ, Brazil | Southeast | Hap07 | Hap07 |
| Ss75 | - | *S. schenckii* | Rio de Janeiro, RJ, Brazil | Southeast | Hap08 | Hap03 |
| Ss78 | - | *S. schenckii* | Rio de Janeiro, RJ, Brazil | Southeast | Hap04 | Hap01 |
| Ss79 | - | *S. brasiliensis* | Rio de Janeiro, RJ, Brazil | Southeast | Hap13 | Hap09 |
| Ss80 | CBS 132969 | *S. schenckii* | Rio de Janeiro, RJ, Brazil | Southeast | Hap09 | Hap08 |
| Ss82 | CBS 132992 | *S. brasiliensis* | Rio de Janeiro, RJ, Brazil | Southeast | Hap13 | Hap09 |
| Ss87 | CBS 132993 | *S. brasiliensis* | Rio de Janeiro, RJ, Brazil | Southeast | Hap13 | Hap09 |
| Ss90 | - | *S. schenckii* | Rio de Janeiro, RJ, Brazil | Southeast | Hap01 | Hap13 |
| Ss111 | CBS 132971 | *S. schenckii* | São Paulo, SP, Brazil | Southeast | Hap05 | Hap02 |
| Ss125 | - | *S. brasiliensis* | Campinas, SP, Brazil | Southeast | Hap16 | Hap09 |
| Ss128 | - | *S. brasiliensis* | Campinas, SP, Brazil | Southeast | Hap17 | Hap09 |
| Ss132 | CBS 132927 | *S. mexicana* | São Paulo, SP, Brazil | Southeast | Hap21 | Hap19 |
| Ss133 | CBS 132928 | *S. mexicana* | Recife, PE, Brazil | Northeast | Hap21 | Hap19 |
| Ss143 | - | *S. schenckii* | Belém, PA, Brazil | North | Hap3 | Hap13 |
| Ss149 | - | *S. brasiliensis* | Pelotas, RS, Brazil | South | Hap13 | Hap11 |
| Ss150 | - | *S. brasiliensis* | Pelotas, RS, Brazil | South | Hap13 | Hap11 |
| Ss151 | CBS 132994 | *S. brasiliensis* | Pelotas, RS, Brazil | South | Hap13 | Hap11 |
| Ss152 | CBS 132995 | *S. brasiliensis* | Pelotas, RS, Brazil | South | Hap13 | Hap11 |
| Ss153 | CBS 132996 | *S. brasiliensis* | Pelotas, RS, Brazil | South | Hap13 | Hap11 |
| Ss154 | - | *S. brasiliensis* | Pelotas, RS, Brazil | South | Hap13 | Hap11 |
| Ss155 | - | *S. brasiliensis* | Pelotas, RS, Brazil | South | Hap13 | Hap11 |
| Ss156 | CBS 132997 | *S. brasiliensis* | Pelotas, RS, Brazil | South | Hap14 | Hap09 |
| Ss157 | CBS 132998 | *S. brasiliensis* | Pelotas, RS, Brazil | South | Hap13 | Hap11 |
| Ss171 | CBS 132999 | *S. brasiliensis* | Londrina, PR, Brazil | South | Hap14 | Hap09 |
| Ss172 | CBS 133000 | *S. brasiliensis* | Londrina, PR, Brazil | South | Hap14 | Hap09 |
| Ss173 | CBS 133001 | *S. brasiliensis* | Londrina, PR, Brazil | South | Hap14 | Hap09 |
| Ss174 | CBS 133002 | *S. brasiliensis* | Londrina, PR, Brazil | South | Hap14 | Hap09 |
| CBS 120339^T^ | CBS 120339^T^ | *S. brasiliensis* | Rio de Janeiro, RJ, Brazil | Southeast | Hap13 | Hap09 |
| IPEC 16919 | IPEC 16919 | *S. brasiliensis* | Rio de Janeiro, RJ, Brazil | Southeast | Hap13 | Hap09 |
| CBS 120340^T^ | CBS 120340^T^ | *S. globosa* | Spain | Spain | Hap12 | Hap16 |
| CBS 130104 | CBS 130104 | *S. globosa* | Spain | Spain | Hap12 | Hap16 |
| CBS 120342 | CBS 120342 | *S. mexicana* | Mexico | Mexico | Hap21 | Hap20 |
| CBS 120341^T^ | CBS 120341^T^ | *S. mexicana* | Mexico | Mexico | Hap21 | Hap20 |
| CBS 302.73^T^ | CBS 302.73^T^ | *S. pallida* | United Kingdom | United Kingdom | Hap22 | Hap21 |
| CBS 111110 | CBS 111110 | *S. pallida* | Germany | Germany | Hap22 | Hap22 |
| CBS 359.36^T^ | CBS 359.36^T^ | *S. schenckii* | USA | USA | Hap01 | Hap13 |
| CBS 937.72^T^ | CBS 937.72^T^ | *S. luriei* | Africa | South Africa | Hap19 | Hap18 |
| Ss226 | CBS 133003 | *S. brasiliensis* | São Paulo, SP, Brazil | Southeast | Hap14 | Hap09 |
| Ss227 | CBS 133004 | *S. brasiliensis* | São Paulo, SP, Brazil | Southeast | Hap13 | Hap09 |
| Ss236 | CBS 132925 | *S. globosa* | Minas Gerais, MG, Brazil | Southeast | Hap12 | Hap10 |
| Ss245 | CBS 133005 | *S. brasiliensis* | Rio de Janeiro, RJ, Brazil | Southeast | Hap13 | Hap09 |
| Ss246 | - | *S. brasiliensis* | Rio de Janeiro, RJ, Brazil | Southeast | Hap13 | Hap09 |
| Ss247 | CBS 133006 | *S. brasiliensis* | Rio de Janeiro, RJ, Brazil | Southeast | Hap13 | Hap09 |
| Ss248 | CBS 133007 | *S. brasiliensis* | Rio de Janeiro, RJ, Brazil | Southeast | Hap13 | Hap09 |
| Ss249 | CBS 133008 | *S. brasiliensis* | Rio de Janeiro, RJ, Brazil | Southeast | Hap13 | Hap09 |
| Ss250 | CBS 133009 | *S. brasiliensis* | Rio de Janeiro, RJ, Brazil | Southeast | Hap13 | Hap09 |
| Ss251 | CBS 133010 | *S. brasiliensis* | Rio de Janeiro, RJ, Brazil | Southeast | Hap13 | Hap09 |
| Ss252 | CBS 133011 | *S. brasiliensis* | Rio de Janeiro, RJ, Brazil | Southeast | Hap13 | Hap09 |
| Ss253 | CBS 133012 | *S. brasiliensis* | Rio de Janeiro, RJ, Brazil | Southeast | Hap13 | Hap09 |
| Ss254 | CBS 133013 | *S. brasiliensis* | Rio de Janeiro, RJ, Brazil | Southeast | Hap13 | Hap09 |
| Ss255 | CBS 133014 | *S. brasiliensis* | Rio de Janeiro, RJ, Brazil | Southeast | Hap13 | Hap09 |
| Ss256 | CBS 133015 | *S. brasiliensis* | Rio de Janeiro, RJ, Brazil | Southeast | Hap13 | Hap09 |
| Ss257 | CBS 133016 | *S. brasiliensis* | Rio de Janeiro, RJ, Brazil | Southeast | Hap13 | Hap09 |
| Ss258 | CBS 133017 | *S. brasiliensis* | Rio de Janeiro, RJ, Brazil | Southeast | Hap13 | Hap09 |
| Ss259 | CBS 133018 | *S. brasiliensis* | Rio de Janeiro, RJ, Brazil | Southeast | Hap13 | Hap09 |
| Ss260 | CBS 133019 | *S. brasiliensis* | Pelotas, RS, Brazil | South | Hap13 | Hap12 |
| Ss261 | - | *S. brasiliensis* | Pelotas, RS, Brazil | South | Hap13 | Hap11 |
| Ss265 | CBS 133020 | *S. brasiliensis* | Uberaba, MG, Brazil | Southeast | Hap20 | Hap09 |
| CBS93872 | CBS 93872 | *S. schenckii* | France | France | Hap02 | Hap14 |
| FMR 8598 | CBS130116 | *S. globosa* | Spain | Spain | Hap12 | Hap10 |
| CMW 304 | CBS 141.36^T^ | *G. serpens* | Italy | Italy | - | - |
| AFTOL-ID 910 | CBS 158.74 | *O. piliferum* | Chile | Chile | - | - |

IPEC, Instituto de Pesquisa Clínica Evandro Chagas, Fiocruz, Brazil; FMR, Facultat de Medicina i Ciències de la Salut, Reus, Spain; CBS, Centraalbureau voor Schimmelcultures, Utrecht, The Netherlands; KMU, Kanazawa Medical University, Ishikawa, Japan; CMW, Culture Collection of the Forestry and Agricultural Biotechnology Institute (FABI); AFTOL, Assembling the Fungal Tree of Life project; NK, not known; ^T^, type strain. All “Ss” strains belong to the culture collection of Federal University of São Paulo (UNIFESP). MG, Minas Gerais; RS, Rio Grande do Sul; PR, Paraná; SP, São Paulo; RJ, Rio de Janeiro; ES, Espírito Santo; PA, Pará; CE, Ceará; GO, Goiás, PE, Pernambuco.
